# Primary small cell carcinoma of the esophagus: Comparison between a Chinese cohort and Surveillance, Epidemiology, and End Results (SEER) data

**DOI:** 10.1002/cam4.2001

**Published:** 2019-02-10

**Authors:** Qin Xiao, Haifan Xiao, Shuyu Ouyang, Jinming Tang, Baihua Zhang, Hui Wang

**Affiliations:** ^1^ Key Laboratory of Translational Radiation Oncology, Department of Radiation Oncology, Hunan Cancer Hospital, The Affiliated Cancer Hospital of Xiangya School Of Medicine Central South University Hunan Province Changsha China; ^2^ Cancer prevention office, Hunan Cancer Hospital, The Affiliated Cancer Hospital of Xiangya School Of Medicine Central South University Hunan Province Changsha China; ^3^ The 2nd Department of thoracic surgery, Hunan Cancer Hospital, The Affiliated Cancer Hospital of Xiangya School Of Medicine Central South University Hunan Province Changsha China

**Keywords:** disparities, multimodal treatment, Primary small cell carcinoma of the esophagus, prognostic factors, SEER

## Abstract

**Background:**

The optimal standard treatment for primary small cell carcinoma of the esophagus (SCCE) remains undetermined. In this study, we conducted two areas of research on SCCE. First, we analyzed differences in SCCE characteristics between Chinese and U.S. patients. Second, we evaluated optimal treatment strategies for SCCE in the Chinese cohort.

**Methods:**

Data from 137 Chinese SCCE patients collected from two cancer centers in China were compared with 385 SCCE patients registered in the U.S. SEER program. Prognostic factors were further analyzed in the Chinese group. Propensity score matching (PSM) was used to balance baseline features between the groups.

**Results:**

There were more Chinese SCCE patients with regional stage disease (41.6%) and surgery was the principal local therapy (78.1%), while 51.7% of U.S. patients was at advanced stages and tended to receive radiotherapy as the main therapy (45.2%). Median overall survival (MST) of Chinese patients was 15.0 months, compared with 8.0 months for U.S. patients (*P *< 0.001). However, the survival differences between groups disappeared after PSM (MST: 12.5 m vs 9.0 m, *P *= 0.144). Further analysis found that surgery tended to achieve clinical benefits only for patients with localized disease (T1‐4aN0M0). Radiotherapy and chemotherapy may prolong survival in patients with regional and extensive disease.

**Conclusions:**

Although there are huge differences in the tumor characteristics and treatment modalities of SCCE between Chinese and U.S. patients, the prognosis of SCCE is equally poor in both. Surgery should be considered for patients with localized disease, while chemoradiotherapy is recommended for patients with regional and extensive disease.

## 1 INTRODUCTION

Primary small cell carcinoma of the esophagus (SCCE) is a highly aggressive malignancy with early metastasis and a dismal prognosis.^1^, ^2^, ^3^, ^4^, ^5^ Because of the rarity of the disease, the optimal standard treatment for SCCE remains undetermined. Furthermore, research on the disparities of the demographics and tumor characteristics of SCCE among different worldwide populations is also scant.

Previous studies consisted of mostly small retrospective series from single countries, and prospective randomized trials seem to be impossible in the near future. However, few studies have been performed based on large databases,^4^, ^5^ such as the Surveillance, Epidemiology, and End Results (SEER) database, which is a population‐based cancer registry covering 28% of the population in the United States. Such large databases have provided a good resource for SCCE research, though there are still limitations because of the incomplete radiochemotherapy information held in the SEER database.

In this article, SCCE data from the U.S. SEER program from 1990 to 2013, and a Chinese cohort consisted of patients diagnosed at Hunan Cancer Hospital, and the Cancer Institute and Hospital, Chinese Academy of Medical Sciences from 1990 to 2012, were collected and systemically compared. We aimed to analyze differences between the two populations regarding demographics and tumor characteristics of SCCE. We also evaluated the optimal treatment strategies and relevant prognostic factors of SCCE in the Chinese cohort.

## 2 MATERIALS AND METHODS

### 2.1 Chinese patient selection

Chinese patients were included in Group ONE in the present study. All patients were initially diagnosed as primary SCCE at Hunan Cancer Hospital and the Cancer Institute and Hospital, Chinese Academy of Medical Sciences from January 1990 to December 2012. All diagnoses of SCCE were confirmed via examination of morphological characteristics and immunohistochemical staining. A total of 143 patients were identified, and six patients were excluded because of incomplete medical records or death or comorbidity in hospital during the first treatment period. The detailed demographic and clinicopathological information of the remaining 137 patients was retrospectively retrieved from the medical records. Either positron emission tomography/computed tomography (PET/CT) or contrast‐enhanced computed tomography (CT) of the chest, Doppler ultrasound, or CT of the abdomen, bone scan, and brain imaging (CT or magnetic resonance imaging (MRI)) were performed to complete the clinical staging evaluation. There was smoking history in 95 patients (69.3%). The American Joint Committee on Cancer (AJCC) 7th edition TNM classification of esophageal carcinoma and the Veterans’ Administration Lung Study Group (VALSG) stage were both used for tumor staging. The study was approved by the Ethics Committee of Hunan Cancer Hospital. All patients provided written informed consent before initiation of the study.

### 2.2 Patient selection in the SEER database

Patients identified in the SEER database were included in Group TWO for further analysis. The SEER database was searched from 1973 to 2015 using site‐specific histology and behavior codes (C15 and malignant). In total, there were 87 864 patients with esophageal malignancy, among which 530 patients (0.6%) with small cell histology were identified using codes (8041, 8042, 8043, and 8045). To avoid selection biases, 385 patients between 1990 and 2013 were selected. Demographic and clinical information was collected, but information regarding chemotherapeutic agents and radiotherapeutic doses was not available for analysis. The VALSG staging system was used for tumor staging, and limited disease was divided into two groups: localized and regional disease. Localized disease was defined as a tumor limited to the primary organ without lymph node metastasis (T1‐4aN0M0); regional disease was defined as a tumor invading directly into surrounding organs or tissues and/or with regional lymph node metastasis (T4b/N+, M0), while extensive (distant) disease was defined as a tumor extended to distant lymph nodes or organs (M1).

### 2.3 Treatment

In Group ONE, curative esophagectomy was performed in 107 patients, including radical resection in 92 cases, R1 resection in two cases, R2 resection in eight cases, and explorative surgery in five cases. The stomach was used as the esophageal substitute, and lymph nodes in the mid and inferior mediastinum and upper abdomen were routinely dissected. Radiotherapy was performed in 44 cases, including postoperative adjuvant radiotherapy in 19 cases, and radical or palliative radiotherapy in 25 cases. A median dose of 60.0 Gy (range, 28‐70 Gy) using 6‐MV photons at 1.8‐2.0 Gy per fraction was delivered. Chemotherapy was administered to 104 patients, including postoperative adjuvant chemotherapy in 67 cases. Combination chemotherapy based on platinum regimens (cisplatin or carboplatin) was the main choice, with a median course of four cycles (range, 1‐12 cycles).

However, considering the limitations of the SEER therapy data, treatment modalities including surgery, chemotherapy, and radiotherapy in Group TWO were not further analyzed.

### 2.4 Statistical analysis

Overall survival (OS) was numbered by months from the beginning of initial treatment to the date of death or last follow‐up. Patients were considered censored if alive or lost to follow‐up at the end of the study. Five‐year OS was considered as the primary endpoint of the analysis.

Differences between groups in demographic and clinicopathological characteristics were assessed by the chi‐square (χ2) test or Wilcoxon rank sum test. OS and median survival time (MST) were analyzed using the Kaplan‐Meier method, and differences between groups were determined by the log‐rank test. The Cox proportional hazard regression model was used to determine the independent prognostic factors and to calculate the hazard ratio (HR). To balance the baseline characteristics between Group ONE and Group TWO, propensity score matching (PSM) with a ratio of 1:1 was performed using the nearest neighbor method, without replacement. The logistic regression model included age, sex, and VALSG stage. *P*‐values of less than 0.05 were considered statistically significant (two‐sided). The statistical package SPSS software version 23.0 (Chicago, IL, USA) was used for statistical analyses.

## 3 RESULTS

### 3.1 Patient characteristics

All patients in Group ONE were of the Han race, while there were 304 Caucasians, 58 African Americans, and 23 people of other races in Group TWO. The characteristics of the two cohorts are summarized in Table 1. Group ONE consisted of 101 (73.7%) men and 36 (26.3%) women, with a mean age of 59.3 years (range, 36‐83 years). However, the proportion of women in Group TWO was relatively higher (38.2%, *P* = 0.012), and the mean age was 69.2 years (range, 41‐94 years) (*P < *0.001). As to the primary tumor locations, the most common location in Group ONE was the mid‐third esophagus (60.6%), while it was the lower third esophagus in Group TWO (44.9%, *P < *0.001). The percentage of combined SCCE histology in Group ONE was also higher than that in Group TWO (14.6% vs 4.7%, *P < *0.001). There were also differences in tumor stage at initial diagnosis; regional disease was the most frequent stage in Group ONE (41.6%), while the majority of Group TWO presented with extensive disease (51.7%, *P < *0.001). There were huge differences in treatment modalities for SCCE between China and the United States. In the Chinese group (ONE), surgery was the main treatment (78.1%), while the proportion of surgery performed in Group TWO was only 8.1% (*P < *0.001). Radiotherapy was the principal local therapy in Group TWO (45.2%), which was performed only in 32.8% of all patients in Group ONE (*P* = 0.012). Although there was a difference in frequency between Group ONE and Group TWO (76.6% vs 66.2%, *P* = 0.024), chemotherapy was the main systemic treatment in both groups. Considering the incompleteness of the radiotherapy and chemotherapy registry in the SEER database, the real percentage of these two therapeutic methods might be higher than reported in Group TWO.

**Table 1:** Demographic and clinical characteristics of Chinese and U.S. SCCE patients

	Group ONE (Chinese)			Group TWO (United States)		*P* value
	No.	Percent		No.	Percent	
Age						
Mean ± SEM	59.3 ± 9.4			69.2 ± 11.5		<0.001
Gender						
Male	101		73.7%	238	61.8%	
Female	36		26.3%	147	38.2%	0.012
Tumor Location						
Cervical esophagus	0		0%	7	1.8%	
Upper third esophagus	19		13.9%	25	6.5%	
Mid‐third esophagus	83		60.6%	98	25.5%	
Lower third esophagus	35		25.5%	173	44.9%	
Undetermined/others	0		0%	82	21.3%	<0.001
Pathological subtype						
Pure SCCE	117		85.4%	367	95.3%	
Combined SCCE	20		14.6%	18	4.7%	<0.001
VALSG stage						
Localized	37		27.0%	64	16.6%	
Regional	57		41.6%	72	18.7%	
Extensive	43		31.4%	199	51.7%	
Unstaged	0		0%	50	13.0%	<0.001
Surgery						
No	30		21.9%	354	91.9%	
Yes	107		78.1%	31	8.1%	<0.001
Radiotherapy						
No	92		67.2%	211	54.8%	
Yes	45		32.8%	174	45.2%	0.012
Chemotherapy						
No	32		23.4%	130	33.8%	
Yes	105		76.6%	255	66.2%	0.024

SEM, standard error of mean; VALSG, the Veterans’ Administration Lung Study Group; SCCE, small cell carcinoma of the esophagus.

### 3.2 Survival analysis and comparison

**Figure 1:**
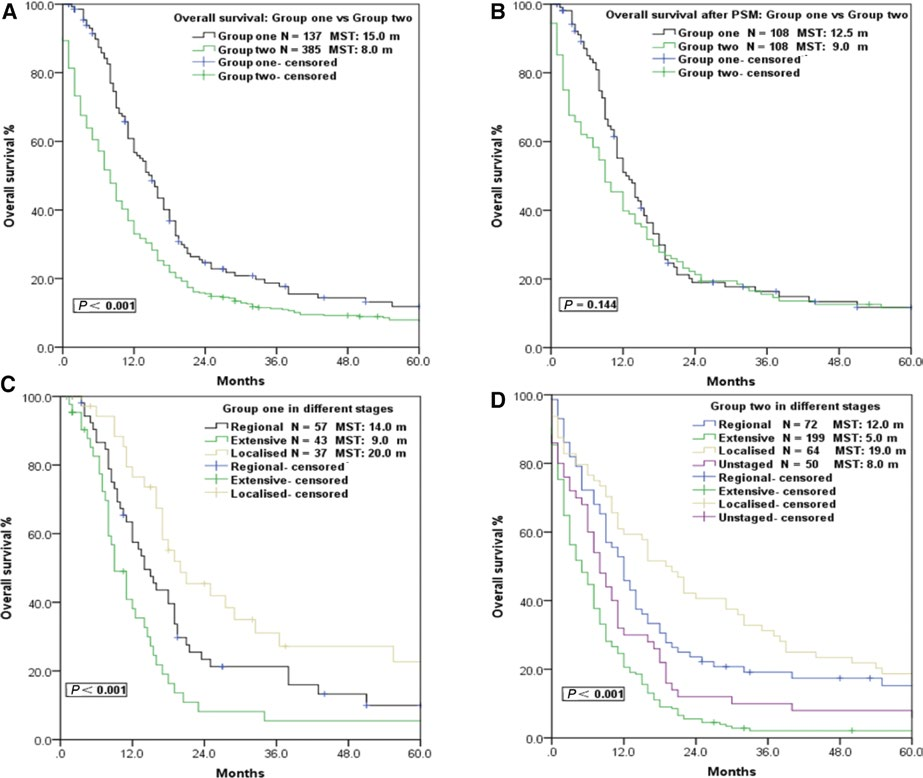
Survival curves of patients in different groups and different VALSG stages. (A) Survival curve comparison between Group ONE and Group TWO. (B) After propensity score matching (PSM), the overall survival differences between 108 patients in Group ONE and those in Group TWO disappeared. (C) Survival curves of different VALSG stages in Group ONE. (D) Survival curves of different VALSG stages in Group TWO

Until December 2015, the median follow‐up of Group ONE was 12.5 months (range, 1‐193 months), while the median follow‐up was 8.0 months for Group TWO. MST and 5‐year OS are shown in Table 2. As illustrated in Figure 1, the MST and 5‐year OS of Group ONE were 15.0 months and 11.9%, respectively, compared with 8.0 months and 7.7%, respectively, for Group TWO (*P* < 0.001). Further analysis demonstrated that VALSG stage was related to OS in both groups, and the MST of localized disease in Group ONE and TWO was 20.0 m and 19.0 m, respectively (Figure 1). Age was not associated with prognosis in Group ONE, while patients aged ≤60 showed better survival in Group TWO (Table 2). However, when PSM was performed and baseline characteristics including age, sex, and VALSG stage were well balanced (Table S1), the overall survival differences between 108 patients in Group ONE and those in Group TWO disappeared (MST: 12.5 m vs 9.0 m, *P* = 0.144; Figure 1).

**Table 2:** Overall survival (OS) analysis of Chinese and U.S. SCCE patients

	Group ONE (n = 137)				Group TWO (n = 385)			
	No.	MST (m, 95% CI)	5‐year OS (%)	*P* value	No.	MST (m, 95% CI)	5‐year OS (%)	*P* value
Age								
≤60	74	16.0 (13.2‐18.8)	11.1		101	11.0 (6.9‐15.1)	11.8	
>60	63	12.5 (8.2‐16.8)	13.2	0.513	284	7.0 (5.7‐8.3)	6.1	0.001
Gender								
Male	101	14.5 (10.9‐18.1)	11.9		238	8.0 (6.7‐9.3)	8.1	
Female	36	15.5 (12.6‐18.4)	10.9	0.832	147	8.0 (6.2‐9.8)	7.0	0.756
VALSG stage								
Localized	37	20.0 (10.9‐29.1)	22.7		64	19.0 (13.5‐24.5)	18.8	
Regional	57	14.0 (10.5‐17.5)	10.0		72	12.0 (9.2‐14.8)	15.2	
Extensive	43	9.0 (6.5‐11.5)	5.4	<0.001	199	5.0 (3.2‐6.8)	2.1	
Unstaged					50	8.0 (13.5‐24.5)	6.0	<0.001
Pathological subtype								
Pure SCCE	117	14.0 (11.2‐16.8)	12.0		367	8.0 (6.9‐9.1)	8.0	
Combined SCCE	20	17.0 (15.7‐18.3)	13.8	0.331	18	9.0 (6.2‐11.8)	0	0.606
Surgery								
Nonradical/no	45	12.0 (7.5‐16.5)	9.5		354	7.0 (6.0‐8.0)	7.2	
Radical	92	17.0 (14.6‐19.4)	13.2	0.046	31	16.0 (11.7‐20.3)	12.9	0.041
Radiotherapy								
No	92	12.0 (8.5‐15.5)	9.2		211	5.0 (3.5‐6.5)	2.4	
Yes	45	17.0 (13.2‐20.8)	16.0	0.172	174	12.0 (9.1‐14.9)	14.1	<0.001
Chemotherapy								
No	32	9.0 (7.5‐10.5)	7.6		130	2.0 (1.4‐2.6)	0	
Yes	105	15.5 (13.1‐17.9)	13.0	0.016	255	11.0 (9.3‐12.7)	11.7	<0.001
Tumor location								
Upper third	19	17.0 (11.6‐22.4)	27.5		25	9.0 (7.1‐10.9)	5.3	
Mid‐third	83	15.5 (11.8‐19.2)	7.1		98	9.0 (6.7‐11.3)	7.8	
Lower third	35	12.0 (6.6‐17.4)	14.6	0.340	173	8.0 (6.2‐9.8)	10.1	0.904
Primary Tumor length								
<5 cm	57	18.0 (15.7‐20.3)	13.6					
≥5 cm	80	11.0 (8.3‐13.7)	10.8	0.022				
T stage								
T1/2	50	18.0 (14.2‐21.8)	11.5					
T3/4	87	13.0 (10.6‐15.4)	11.8	0.082				
N stage								
N‐	37	20.0 (10.9‐29.1)	22.7					
N+	100	12.0 (9.7‐14.3)	8.3	0.001				
TNM stage								
I/IIA	35	19.0 (13.9‐24.1)	21.7					
IIB	22	18.0 (12.8‐23.2)	13.9					
III/IV	80	11.0 (8.5‐13.5)	9.5	0.013				
Family history								
No	116	15.0 (12.5‐17.5)	12.6					
Yes	19	15.0 (10.1‐19.9)	9.1	0.804				
Multimodal treatment								
Local	31	9.0 (7.6‐10.4)	11.8					
Systemic	11	8.0 (5.8‐10.2)	0					
Local+systemic	95	17.0 (14.6‐19.4)	14.2	<0.001				

SCCE, small cell carcinoma of the esophagus; VALSG, the Veterans’ Administration Lung Study Group; Local, surgery/radiotherapy; Systemic, chemotherapy.

### 3.3 Treatment and survival

**Figure 2:**
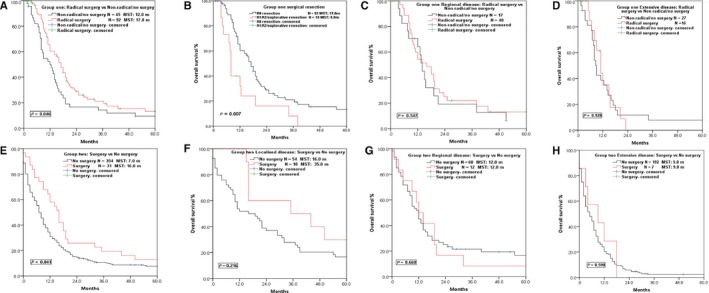
Survival curves of patients in different groups and different VALSG stages who received surgery or not

Stratified analysis was performed to evaluate the impact of treatment modalities on the prognosis of SCCE. The MST of patients who received radical resection in Group ONE was 17.0 m, which was longer than that of patients who received nonradical or no surgery (12.0 m, *P* = 0.046; Figure 2), in accordance with the result in Group TWO (MST: 16.0 m vs 7.0 m, *P* = 0.041; Figure 2). Patients received R0 resection in Group ONE also showed better survival than R1/R2 or explorative resection (MST: 17.0 m vs 8.0 m, *P* = 0.007; Figure 2). In the subgroup of localized disease in Group TWO, patients who received surgery generally survived longer than patients who received no surgery and the survival curves were well separated, but no statistical significant differences were identified (Figure 2). However, in the subgroups of regional disease and extensive disease, no survival benefit was observed in patients who received surgery or not in both groups (Figure 2).

**Figure 3:**
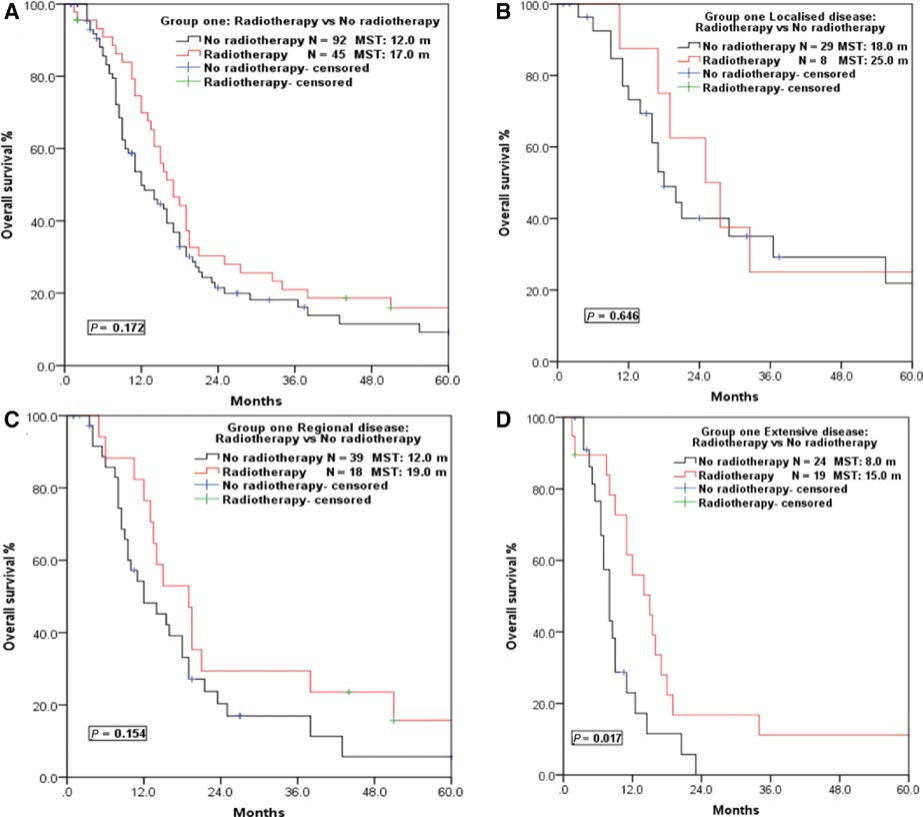
Survival curves of Group ONE patients in different VALSG stages who received radiotherapy or not. (A) Survival curves of patients who received radiotherapy or not in Group ONE. (B) Survival curves of patients with localized disease who received radiotherapy or not. (C) Survival curves of patients with regional disease who received radiotherapy or not. (D) Survival curves of patients with extensive disease who received radiotherapy or not

Considering the incompleteness of radiochemotherapy information in the SEER database, the analysis of survival benefits for radiochemotherapy was performed only in Group ONE. As illustrated in Figure 3, patients in Group ONE who received radiotherapy exhibited a relatively longer MST than patients who did not receive radiotherapy (17.0 m vs 12.0 m, *P* = 0.172), which was in accordance with the result of Group TWO (MST 12.0 m vs 5.0 m, *P* < 0.001; Table 2). Subgroup analysis based on VALSG stages revealed that greater survival benefit was achieved via radiotherapy in patients with regional (MST 19.0 m vs 12.0 m, *P* = 0.154) and extensive disease (MST 15.0 m vs 8.0 m, *P* = 0.017) but not localized disease (MST 25.0 m vs 18.0 m, *P* = 0.646) (Figure 3).

**Figure 4:**
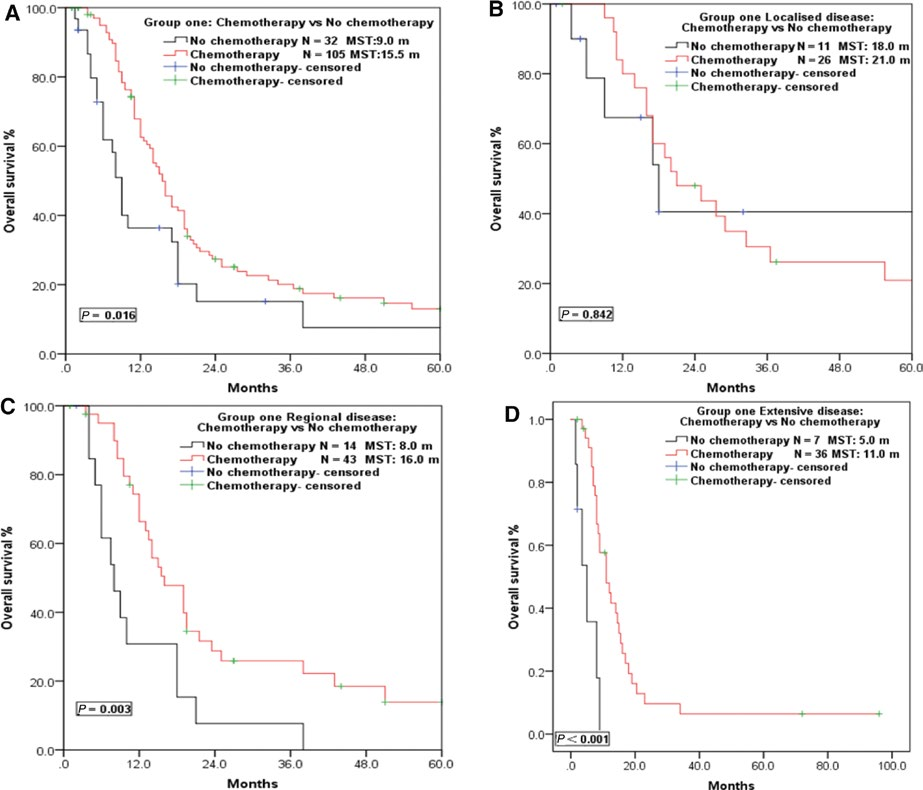
Survival curves of Group ONE patients in different VALSG stages who received chemotherapy or not. (A) Survival curves of patients who received chemotherapy or not in Group ONE. (B) Survival curves of patients with localized disease who received chemotherapy or not. (C) Survival curves of patients with regional disease who received chemotherapy or not. (D) Survival curves of patients with extensive disease who received chemotherapy or not

**Figure 5:**
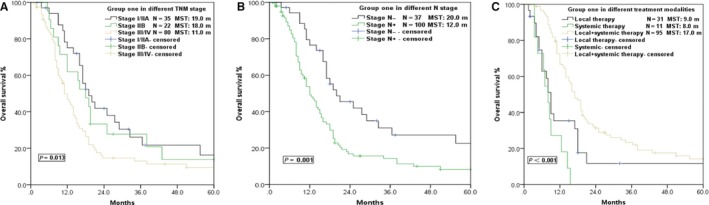
Survival curves of Group ONE patients in different TNM stages who received different treatment modality. (A) Survival curves of patients with different TNM stages. (B) Survival curves of patients with or without regional lymphatic metastasis. (C) Survival curves of patients who received local therapy (radiotherapy/surgery) + systemic therapy (chemotherapy), local therapy, or systemic therapy alone

Chemotherapy was the mainstay of treatment in both groups. The MST of patients received chemotherapy in Group ONE was 15.5 m, significantly longer than the 9.0 m in patients who received no chemotherapy (*P* = 0.016) (Figure 4), which was in line with the result for Group TWO (MST 11.0 m vs 2.0 m, *P* < 0.001; Table 2). Further stratified analysis indicated that patients with regional (MST 16.0 m vs 8.0 m, *P* = 0.003) and extensive disease (MST 11.0 m vs 5.0 m, *P* < 0.001) would obtain a survival benefit via chemotherapy, but no survival differences were observed in patients with localized disease (MST 21.0 m vs 18.0 m, *P* = 0.842; Figure 4). Furthermore, patients who received local therapy (radiotherapy/surgery) combined with systemic therapy (chemotherapy) showed the best long‐term survival, with a MST and 5‐year OS of 17.0 m and 14.2%, respectively, compared with patients who only received local therapy or systemic therapy alone (MST: 8.0, 9.0 m, *P* < 0.001; Figure 5).

### 3.4 Prognostic factors analysis in Group ONE

Univariate analysis of prognosis for the 137 SCCE patients in Group ONE is summarized in Table 2. The MST and 5‐year OS were 18.0 m and 13.6%, respectively, in patients with tumors <5 cm in diameter, and 11.0 m and 10.8%, respectively, in patients with tumors ≥5 cm (*P* = 0.022). The MST of patients with stage I/IIA and IIB tumors was longer than that of those with stage III/IV tumors, at 19.0 m, 18.0 m, and 11.0 m, respectively (Figure 5). The MST and 5‐year OS of patients without regional lymphatic metastasis (N‐) were 22.0 m and 22.7%, respectively, which was also longer than that with lymphatic metastasis (N+), at 12.0 m and 8.3%, respectively (*P* = 0.001; Figure 5). However, other factors including age, sex, pathological subtype, radiotherapy, T stage, tumor location, and family history of malignancy were not associated with the prognosis of SCCE in Group ONE.

The results of the multivariate analysis are shown in Table 3. Factors included in the Cox proportional hazards model were primary tumor length, surgery, TNM stage, VALSG stage, N stage, chemotherapy, and multimodal treatment. The VALSG stage (HR, 1.793), N stage (HR, 8.473), and multimodal treatment (HR, 0.400) were found to be possible independent prognostic factors for SCCE.

**Table 3:** Multivariate survival analysis of 137 SCCE patients in Group ONE

Variable	HR	95% CI	*P* value
Primary Tumor length			
＜5 cm vs ≥5 cm	1.371	0.849‐2.213	0.197
Surgery			
Radical vs Nonradical/no	1.051	0.618‐1.788	0.853
TNM stage			
I/IIA vs IIB vs III/IV	0.877	0.650‐1.184	0.393
VALSG stage			
Localized vs Regional vs Extensive	1.793	1.076‐2.988	0.025
N stage			
N‐ vs N+	8.473	1.984‐36.185	0.004
Chemotherapy			
Yes vs No	2.188	0.447‐10.712	0.334
Multimodal treatment			
Local vs Systemic vs Local+systemic	0.400	0.183‐0.874	0.022

SCCE, small cell carcinoma of the esophagus; VALSG, the Veterans’ Administration Lung Study Group; Local, surgery/radiotherapy; Systemic, chemotherapy.

## 4 DISCUSSION

Few studies have reported discrepancies among the demographics, tumor characteristics, and treatment modalities of SCCE in different ethnic groups. To the best of our knowledge, our study is the first to demonstrate that there are huge differences in the tumor characteristics and treatment modalities of SCCE between patients in China and the United States. Interestingly, the proportion of female patients was relatively higher, and the mean age at diagnosis was 10 years older in the U.S. group than in the Chinese group. The most common primary location was the lower third esophagus in the U.S. group, while it was the mid‐third esophagus in the Chinese group. There were more Chinese SCCE patients with earlier stage disease and surgery was the main local therapy, while approximately half of the U.S. patients were at advanced stages and tended to receive radiotherapy as the principal local therapy. However, further stratified analyses revealed that surgery could achieve clinical benefits only for patients with localized disease both in the U.S. and Chinese groups, while radiotherapy and chemotherapy might be able to prolong survival in patients with regional and extensive disease.

Esophageal cancer is one of the leading causes of cancer mortality in China, and approximately 90% of all esophageal malignancies are squamous cell carcinoma and are mostly located in the mid‐third esophagus.^6^, ^7^, ^8^, ^9^ However, the United States is one of the low‐incidence regions of esophageal cancer, and the most common pathological type is adenocarcinoma, originating mostly in the lower third of the esophagus.^8^, ^10^ In the present study, in the Chinese SCCE group, patient age, sex, primary tumor location, and tumor length were similar to that of esophageal squamous cell carcinoma, in accordance with the results of previous reports from China.^1^, ^3^, ^11^, ^12^, ^13^, ^14^ In contrast, the clinical features of SCCE patients in the U.S. group were more similar to that of adenocarcinoma.^5^, ^15^, ^16^, ^17^ Such differences indicated that critical etiologic factors including genetic and environmental factors might differ substantially among SCCE patients in China and the United States, just as those unique risk factors that contribute to squamous cell carcinoma and adenocarcinoma .^8^, ^9^, ^15^, ^16^ These differences should be considered for the management of SCCE patients of different ethnic backgrounds.

Previous studies have suggested that esophageal squamous cell carcinoma in developing countries and adenocarcinoma in developed country requires different therapeutic strategies.^10^, ^15^ Although there are huge differences in the demographic and clinical characteristics of patients in the United States and China, the prognosis of SCCE in both populations seems to be equally poor. In our study, the OS of the Chinese group was higher than that of the U.S. group, but the survival difference disappeared after PSM, suggesting that there was no significant difference in the prognosis of SCCE between the two groups if the disparities in patient age, sex, and VALSG stage were removed. Furthermore, previous studies have reported similar pathological features, immunohistochemical staining, and aggressive nature of SCCE among different worldwide populations.^1^, ^3^, ^17^, ^18^, ^19^, ^20^, ^21^, ^22^^,^ 23 ^,^ 24 ^,^ 25 Therefore, we concluded that SCCE from different populations should be analyzed as a single entity, though there might be discrepancies in the pathogenesis, demographics, and certain clinical features.

There is a vast discrepancy in the surgical treatment of SCCE in China and the United States, suggesting that the role of surgery in the treatment of SCCE has been controversial until now.^2^, ^18^, ^26^ Approximately 78.1% of all Chinese patients undergo surgery, similar to Japan,^27^ and significantly higher than the 8.1% in the U.S. group. Ku et al^18^ reported that limited‐stage SCCE patients could achieve long‐term survival after induction chemotherapy followed by consolidative chemoradiation. In the study by Lv et al,^2^ no difference in MST and locoregional recurrence was observed between patients who received surgery or not. However, Situ et al^26^ and Xie et al^11^ demonstrated that radical esophagectomy with extended lymphadenectomy should be considered for patients with limited‐stage SCCE. A larger retrospective study based on the National Cancer Data Base proved that esophagectomy improved the survival of patients with localized disease (node‐negative) compared with chemotherapy alone or chemoradiotherapy.^4^ Furthermore, several other studies 1, 3, 28 demonstrated that radical esophagectomy achieved the best survival in patients with stage I/IIA SCCE. In the present study, a stratified analysis based on VALSG stage grouping revealed that radical resection had a tendency to achieve longer survival in patients with localized disease, though this was not statistically significant. In the subgroups of regional disease and extensive disease, no survival difference was observed between patients who received surgery or not in both groups. Therefore, we propose that surgery should be considered only for patients with localized disease (T1‐4aN0M0).

As another local therapeutic approach, radiotherapy was usually considered as a substitute of surgery in several previous studies. Meng et al^29^ reported that the survival of limited‐stage SCCE treated by chemoradiotherapy was better than that treated by surgery followed by chemotherapy. Lv et al^2^ recommended that chemoradiotherapy should be the standard of care for limited‐stage SCCE. In the present study, radiation was the most favored local therapy for the U.S. group and was utilized in 45.2% of patients, achieving better survival than nonradiation‐based treatment. This finding is consistent with the result of the Chinese group, though statistical significance was lacking in the latter group. However, the subgroup analysis was performed only in the Chinese group because incomplete radiochemotherapy information is held in the SEER database. Survival benefits were observed in patients with regional and extensive disease, but not in localized disease. This result is in concordance with several previous results.^4^, ^28^

SCCE is considered a systemic disease and chemotherapy should always be part of the treatment plan.^11^, ^20^, ^21^, ^30^, ^31^ Chemotherapy was a cornerstone in the treatment of SCCE in the present study. Our results showed that chemotherapy improved OS both in the Chinese and U.S. groups. However, in a stratified analysis, chemotherapy failed to improve survival in localized stage patients (T1‐4aN0M0), which was in line with the results of several large‐scale retrospective studies.^1^, ^3^, ^12^ In studies by Xu et al^1^ and Chen et al,^3^ postoperative adjuvant therapy could not improve survival in stage I/IIA patients. Zou et al^12^ reported that postoperative chemotherapy improves survival only in SCCE patients at stages T3‐4N0M0 and T1‐4N1‐2M0. In a meta‐analysis by Raja et al,^19^ adding surgery or radiotherapy to chemotherapy further improved survival. Moreover, concurrent chemoradiotherapy could achieve greater survival benefit for limited disease SCCE compared with a sequential approach.^20^ In the present study, local therapy including surgery and radiotherapy combined with chemotherapy improved survival much more than local therapy or chemotherapy alone. Additionally, multivariate analysis disclosed that stage N‐, localized disease, and multimodal treatment were favorable independent prognostic factors.

There are always inherent limitations with any retrospective study. Radiotherapy doses, regimens and cycles of chemotherapy, and tumor recurrences are not recorded in the SEER database nor are treatments that patients received after initial therapy. Thus, our study was limited to the available variables and many possible factors affecting the disease were not considered. For example, patients’ performance status should also be considered since it might affect treatment modality and survival. Moreover, the Chinese group in our study is not representative of all SCCE patients in China since the sample size is too small and only collected from two cancer centers. Therefore, caution should be applied when interpreting our results. Studies with more comprehensive and larger sample sizes should be performed to obtain a clearer picture of SCCE in the future.

In conclusion, although there are huge differences in the tumor characteristics and treatment modalities of SCCE between patients in China and the United States, the prognosis of SCCE is equally poor in both groups. Surgery should be considered for patients with localized disease (T1‐4aN0M0), and chemotherapy combined with radiotherapy might be recommended for patients with regional and extensive disease. Stage N‐, localized disease, and multimodal treatment are independent favorable prognostic factors.

## CONFLICT OF INTEREST

The authors indicated no potential conflicts of interest.
