# Supplementary material for: Primary small cell carcinoma of the esophagus: Comparison between a Chinese cohort and Surveillance, Epidemiology, and End Results (SEER) data
Source: Cancer Med. 2019 Feb 10;8(3):1074–85. doi: 10.1002/cam4.2001 (PMC6434219; doi:10.1002/cam4.2001)
Supplement: Supplementary file 1 [file CAM4-8-1074-s001.doc]

**Supplemental Table 1. Demographic and clinical characteristics of Chinese and U.S. SCCE patients after PSM.**

|  | Group ONE (n=108) | | | Group TWO (n=108) | |  |
| --- | --- | --- | --- | --- | --- | --- |
|  | No. | percent | | No. | percent | P value |
| Age |  |  | |  |  |  |
| mean±SEM | 61.08±9.00 | | | 61.12±8.96 | | 0.808 |
| Gender |  | |  |  |  |  |
| Male | 79 | | 73.1% | 82 | 75.9% |  |
| Female | 29 | | 26.9% | 26 | 24.1% | 0.639 |
| VALSG stage |  | |  |  |  |  |
| Localised | 22 | | 20.4% | 22 | 20.4% |  |
| Regional | 45 | | 41.7% | 45 | 41.7% |  |
| Extensive | 41 | | 38.0% | 41 | 38.0% | 1.000 |
| PSE, propensity score matching; SEM, standard error of mean; VALSG, the Veterans’ Administration Lung Study Group; SCCE, small cell carcinoma of the esophagus. | | | | | | |
